# Using focus groups to design systems science models that promote oral health equity

**DOI:** 10.1186/s12903-018-0560-0

**Published:** 2018-06-04

**Authors:** Susan S. Kum, Mary E. Northridge, Sara S. Metcalf

**Affiliations:** 1 Department of Geography, The State University of New York at Buffalo, 115 Wilkeson Quad, Ellicott Complex, Buffalo, NY 14261-0055 USA; 2 Department of Population Health, New York University School of Medicine, 227 East 30th Street, 8th Floor, New York, NY 10016 USA; 3 Department of Epidemiology and Health Promotion, New York University College of Dentistry, 433 First Avenue, Room 726, New York, NY 10010 USA; 4 Department of Sociomedical Sciences, Columbia University Mailman School of Public Health, 722 West 168th Street, New York, NY 10032 USA

**Keywords:** Oral public health, Dental public health, Oral health equity, Systems science, Agent-based modeling, Qualitative analysis, Focus group analysis, Racial/ethnic minorities, Older adults, Community-based oral health care

## Abstract

**Background:**

While the US population overall has experienced improvements in oral health over the past 60 years, oral diseases remain among the most common chronic conditions across the life course. Further, lack of access to oral health care contributes to profound and enduring oral health inequities worldwide. Vulnerable and underserved populations who commonly lack access to oral health care include racial/ethnic minority older adults living in urban environments. The aim of this study was to use a systematic approach to explicate cause and effect relationships in creating a causal map, a type of concept map in which the links between nodes represent causality or influence.

**Methods:**

To improve our mental models of the real world and devise strategies to promote oral health equity, methods including system dynamics, agent-based modeling, geographic information science, and social network simulation have been leveraged by the research team. The practice of systems science modeling is situated amidst an ongoing modeling process of observing the real world, formulating mental models of how it works, setting decision rules to guide behavior, and from these heuristics, making decisions that in turn affect the state of the real world. Qualitative data were obtained from focus groups conducted with community-dwelling older adults who self-identify as African American, Dominican, or Puerto Rican to elicit their lived experiences in accessing oral health care in their northern Manhattan neighborhoods.

**Results:**

The findings of this study support the multi-dimensional and multi-level perspective of access to oral health care and affirm a theorized discrepancy in fit between available dental providers and patients. The lack of information about oral health at the community level may be compromising the use and quality of oral health care among racial/ethnic minority older adults.

**Conclusions:**

Well-informed community members may fill critical roles in oral health promotion, as they are viewed as highly credible sources of information and recommendations for dental providers. The next phase of this research will involve incorporating the knowledge gained from this study into simulation models that will be used to explore alternative paths toward improving oral health and health care for racial/ethnic minority older adults.

**Electronic supplementary material:**

The online version of this article (10.1186/s12903-018-0560-0) contains supplementary material, which is available to authorized users.

## Background

Since the end of World War II and the subsequent increase in living standards and community water fluoridation [1], the US population has benefitted from substantial reductions in tooth decay and edentulism (complete tooth loss). The declining prevalence of edentulism in the older adult population means there is greater retention of natural teeth requiring oral health care as people age [2, 3]. Prevention and treatment efforts, technological advancements in dentistry, and dental insurance have also contributed to improved oral health [4]. Yet despite these documented improvements in oral health over time, the major oral diseases—dental caries and periodontal diseases—are still among the most prevalent chronic conditions in the US population across the life span [5].

There remain critical unmet oral health needs among children and adolescents, young and working age adults, and older adults. While the public views oral health as a priority, oral health concerns are inadequately addressed by public policies, especially for older adults [6]. For instance, because the Medicare program in the United States for persons aged 65 years and older and disabled adults does not cover routine dental care, many older adults are unable to afford the necessary preventive and restorative treatments they need [7]. Racial/ethnic minority older adults are at higher risk for edentulism than the white majority population [8]. They have also been found to be less likely to report dental visits in the past year, perhaps due to language barriers, especially among the foreign-born [9]. In addition to experiencing poorer clinical measures of oral health, racial/ethnic minority older adults also report worse self-rated oral health than their white counterparts [10].

To effectively address oral health and health care inequities requires reforming public policies for how oral health services are financed and delivered [6]. There are multiple identified factors operating at different scales that contribute to individual and population oral health inequities [11]. Therefore, strategies are needed to support and advance oral health promotion and treatment initiatives, along with oral health policy changes at local, state, and national levels. These strategies include place-based programs that apply principles of geographic targeting or directed population approaches to promote oral health equity. A primary objective of these strategies is to identify gaps in dental services and population oral health needs. Toward advancing research designed to promote oral health equity, this paper presents an approach to explicating and incorporating the perceptions and knowledge of African American, Dominican, and Puerto Rican older adults related to oral health care in their neighborhoods.

Several conceptual models for studying access to health services have been proposed. For instance, a framework devised by Penchansky and Thomas defines access as the fit between service providers and service users [12]. There are 5 dimensions for which fit is compared: (1) accessibility, i.e., the locations of service providers versus the locations of service users; (2) availability, i.e., the volume and type (supply) of services versus the volume and types of needs; (3) affordability, i.e., the cost of services versus the ability of service users to pay for them; (4) accommodation, i.e., the approach to provision by service providers versus the perceptions of appropriateness by service users; and (5) acceptability, i.e., service users’ reactions to and expectations of service providers versus service providers’ reactions to and expectations of service users [12]. Discrepancies in fit influence use of services, satisfaction of service users, and practices of service providers.

Powell offers an alternative taxonomic definition of access to health services that considers social accessibility to be comprised of 3 dimensions: affordability, accommodation, and acceptability [13]. In the present analyses, the question asked was: “Why do older adults go to a dental provider?” Responses were used to determine aspects of dental providers and oral health care settings that influence older adults’ decisions about where they seek oral health care, i.e., social accessibility.

## Methods

### Qualitative methods

Valuable information for understanding and addressing problems is often held in qualitative forms of data, such as in mental models and written texts [14]. Similarly, the humanistic approach emphasizes the lived experiences and personal histories of individuals [15]. Narratives may provide meaning and context to an individual’s experiences with illness and recovery [16]. Interpreting narratives may reveal characteristics associated with an individual’s health status that cannot otherwise be detected, such as hope, despair, fear, guilt, and grief. Narratives may point to new hypotheses and stimulate more patient-centered research [17].

### Focus group approach and participants for the study

Focus groups were conducted with a sample of 194 racial/ethnic minority men and women aged 50 years and older living in northern Manhattan who participated in one of 24 focus group sessions about improving oral health for older adults [18]. The investigators of the study selected focus groups over individual interviews because group discussions may facilitate greater disclosure by participants through reciprocity, i.e., disclosure by one participant may prompt greater disclosure by others [19]. Further, focus groups allow participants to respond to and elaborate on topics raised by fellow participants, thus facilitating discussion of a greater breadth of topics [19]. Finally, focus groups may be less fatiguing than individual interviews, which may be particularly important when interviewing older adults [20].

Focus group participants had to meet the following criteria: (1) aged 50 years or older; (2) attended a senior center or other community locale where older adults gather in northern Manhattan; (3) speak fluent English or Spanish; and (4) self-identify as African American, Dominican, or Puerto Rican. The demographic characteristics of the focus group participants overall and by gender are presented in Table 1.

**Table 1:** Characteristics of participants in focus groups for the total sample and by gender, New York, NY, 2013–2015

Participants and Focus Groups		Total Sample	Women	Men
Participants		*N* = 194	*n* = 104	*n* = 90
Focus groups		*N* = 24	n = 12	*n* = 12
Characteristics		% (n)	% (n)	% (n)
Age group in years	50–59	14.4% (28)	16.3% (17)	12.2% (11)
	60–69	34.0% (66)	32.7% (34)	35.6% (32)
	70–79	36.1% (70)	34.6% (36)	37.8% (34)
	80–89	11.9% (23)	11.5% (12)	12.2% (11)
	90+	3.6% (7)	4.8% (5)	2.2% (2)
Race/ethnicity	Dominican	35.6% (69)	33.7% (35)	37.8% (34)
	Puerto Rican	27.3% (53)	27.9% (29)	26.7% (24)
	African American	37.1% (72)	38.5% (40)	35.6% (32)
Last dental visit	Within past year	54.1% (105)	52.9% (55)	55.6% (50)
	1–3 years ago	27.3% (53)	31.7% (33)	22.2% (20)
	> 3 years ago	18.6% (36)	15.4% (16)	22.2% (20)
Primary language	English	42.3% (82)	46.2% (48)	37.8% (34)
	Spanish	48.5% (94)	45.2% (47)	52.2% (47)
	Both	9.3% (18)	8.7% (9)	10.0% (9)

Women and men did not differ significantly on any of the characteristics listed above, in accordance with the sampling strategy

### Recruitment procedure, sampling strategy, and context

Field recruitment staff visited senior centers in northern Manhattan and directly approached older adults to explain the study, screen them for eligibility, and solicit participation in the focus groups. Senior centers were selected rather than places where older adults receive dental care in order to obtain a sample of individuals who did not necessarily have access to, or seek, dental care.

To ensure geographic and demographic representation of northern Manhattan, approximately equal numbers of participants were recruited from senior centers in each of three northern Manhattan neighborhoods: Central / West Harlem (home to large numbers of African Americans), Washington Heights / Inwood (home to large numbers of Dominicans), and East Harlem (home to large numbers of Puerto Ricans). These 3 neighborhoods have historically been considered as racial/ethnic enclaves, with large numbers of recent immigrants and many residents qualifying for Medicaid and other forms of public assistance. Further details of the recruitment and screening procedures are available elsewhere [18].

The study design of 24 focus groups was selected a priori in order to obtain multiple groups of each demographic segment, thereby allowing conclusions about each demographic segment to be based on multiple focus group discussions rather than on a single focus group discussion. Consistent with standard focus group techniques [21], the groups were segmented based on important characteristics that may influence the issues discussed or the ability of the members to build rapport. A total of 24 focus groups were conducted, including 12 groups of men and 12 groups of women. Within each gender set, 4 groups were conducted with African Americans, 4 groups were conducted with Dominicans, and 4 groups were conducted with Puerto Ricans. Within each gender / ethnic / racial set, half of the groups were conducted with participants who had visited a dentist in the past year and half were conducted with participants who had not visited a dentist in the past year. Ten groups were conducted in English (including two groups with Puerto Ricans who preferred to speak English) and 14 groups were conducted in Spanish. An average of 8 older adults participated in each of the 24 focus groups.

### Systems science methods

Paina and Peters propose viewing health systems through the lens of complex adaptive systems to offer insights and inform planning, implementing, monitoring, and evaluating more effective, equitable, sustainable, and context-relevant approaches to population health needs and demands [22]. For the study of complex phenomena, systems science provides a variety of research methods that are complementary to qualitative approaches [23]. These include system dynamics, agent-based modeling, social network analysis, and geographic information science [24–28].

Among the findings of a recent systematic review is that soft systems modeling techniques are likely to be the most useful addition to public health [29]. This is because the methodological positioning and subsequent metaphors in systems science, such as feedback, accumulation, and endogenous behavior, provide a way to conceptualize complex and politically sensitive problems and policies of the health system, and in the process, facilitate knowledge transfer among researchers, practitioners, and policymakers [29]. On the other hand, there also needs to be greater accountability in hard systems modeling, especially in terms of explicating the procedures used to build computer models. This would allow for other researchers and practitioners to contribute to the modeling process and assess whether or not the right questions are being addressed. More realistic, data-intensive computer models may lack transparency and flexibility, becoming too complicated and difficult to comprehend to be of practical use [30, 31].

A causal map is a visual artifact used in the field of system dynamics, a scientific approach that involves representing, testing, and modifying assumptions about dynamically complex problems attributed to feedback loops and delays [32]. Causal mapping is a method for articulating a plausible explanation for these dynamically complex problems that is used to identify feedback loops. Causal maps are integral elements in a preliminary blueprint for computer models, and in this research, are considered to be boundary objects [33]. Black defines a boundary object as “a representation—perhaps a diagram, sketch, sparse text, or prototype—that helps individuals collaborate effectively across some boundary, often a difference in knowledge, training, or objective.” ([34], 76, p).

### Integrating qualitative and systems science methods in the data analysis

Importantly, a causal map depicts cause-and-effect relationships, where arrows between variables are used to specify the direction of cause and effect. A polarity is assigned to each relationship indicating whether the effect (variable Y) increases or decreases relative to the cause (variable X) ceteris paribus, i.e., holding all else equal.

A protocol proposed by Kim and Andersen for coding qualitative text data and developing causal maps from purposive text data was adapted here to analyze decisions on where older adults seek oral health care [14]. The initial stage involves critically evaluating the text to extract data segments and cause-and-effect relationships. This corresponds to open coding, i.e., the summarizing and division of text that captures specific phenomena or experiences that may be used as codes. As codes are grouped, dominant patterns or themes emerge and are observed and subsequently coded. The next stage involves the grouping together of similar data segments to arrive at more generalized cause-and-effect relationships and implicit structures in order to construct a composite causal map. This step corresponds to axial coding, i.e., the organization of relationships among categories of codes to establish relationships between categories. Implicit structures, i.e., intermediate variables, guide the merging of categories of codes.

A topic guide consisting of open-ended questions was developed by the research team and used by the focus group moderators to facilitate the sharing of personal experiences among participants (see Additional file 1). The initial categories for organizing the qualitative data were based upon the topic guide; additional categories were included during subsequent readings of the transcripts. Focus groups with content related to categories of interest were indicated and recorded. An iterative process was used to extract excerpts of transcript text, which are referred to as data segments.

Data segments are typically a single response from a participant, i.e., a portion of text indicated in the focus group by “P:” indicating “Participant:” in the transcripts. For certain data segments, it was important to capture the dialogue. Hence, a forward slash (/) is used to indicate multiple responses. Cause-and-effect relationships are then identified from data segments.

Table 2 presents the topics by type (either finding dental providers or going to dental providers) identified from the 24 focus groups.

**Table 2:**
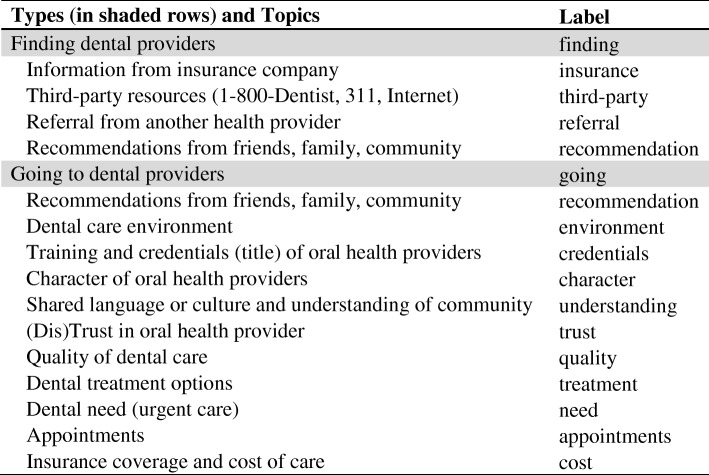
Decisions on where to go for oral health care organized by types and topics

Topics refer to the pattern or theme that was determined by examining a grouping of similar data segments and were identified at the time of extraction of data segments. Labels were then assigned to types and topics to facilitate the filtering of the data segments in a spreadsheet. Next, each data segment was assigned a data segment Type, Topic, and identification number (SegmentID). When assigning SegmentIDs, replicate entries were removed that had been introduced erroneously during data extraction.

## Results

**Fig. 1:**
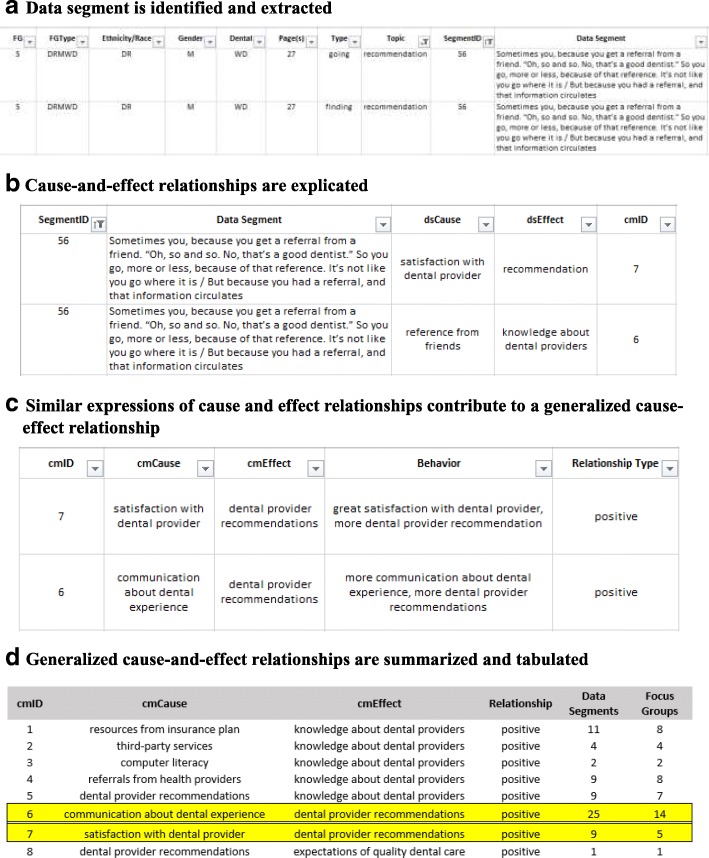
Process by which a data segment is recorded and organized. An illustration of the process by which an extracted data segment is recorded and organized. Specifically, data segment 56 (SegmentID 56) originates from focus group 5 conducted with Dominican men who received oral health care in the past year (translated into English from Spanish). The 4 panels correspond to the following steps: (**a**) Data segment is identified and extracted; (**b**) Cause and effect relationships are explicated; (**c**) Similar expressions of cause and effect relationships contribute to a generalized cause-and-effect relationship; and (**d**) Generalized cause-and-effect relationships are summarized and tabulated

Figure 1 provides an illustration of the process by which a data segment and corresponding focus group segmentation information is recorded and organized.

Certain data segments yielded more than a single cause-and-effect relationship; thus, there are 287 relationships from 240 data segments. The data segment shown in Fig. 1a is SegmentID 56, which was extracted from focus group 5, comprised of Dominican men who had received a dental visit in the past year (translated into English from Spanish). This data segment was used to explicate 2 cause-and-effect relationships: the first for finding dental providers and the second for going to dental providers. This is an especially interesting data segment because it represents both the view of the speaker and the view of his friend.

A data segment cause (dsCause) and a data segment effect (dsEffect) were then identified for each data segment. Attempts were made to extract terms from each data segment to express dsCause and dsEffect. To continue with the illustration, Fig. 1b presents the cause-and-effect relationships identified for SegmentID 56. For data segments where it was difficult to maintain complete data integrity, terms derived from the interpretation of the first author for the data segment were used.

Similar expressions of cause and effect relationships, aided by the filtering of labels, were then generalized into a causal map cause (cmCause) and a causal map effect (cmEffect). For example, Fig. 1c presents the cmCause and cmEffect to which SegmentID 56 contributed. Data segments in the same topic group were compared to derive a term that was representative of the relationships expressed in that subset of data segments. Deliberate attempts were made to use variables that were already assigned; new variables were typically generated to clarify relationships. Each of the generalized relationships was then assembled in Vensim software [35] to provide a visual overview of the existing relationships. Certain of the generalized relationships were collapsed during the process of assembly and intermediate structures were added. The order in which the variables were assigned a cmID, indicating the generalized cause and effect relationships, followed the descending order of topics presented in Table 1. One of the generalized relationships in a topic group would often provide a link to another topic group. To complete the example, Fig. 1d presents an illustration of how the generalized cause-and-effect relationships are summarized and tabulated.

The behavior (noted simply as Behavior) of the cmCause and cmEffect was then assessed. The Behavior was articulated to check the logic of each cause and effect relationship and assign a polarity to that relationship. The polarity of the relationship, that is, whether the behavior of the cause and effect changes in the same direction (positive) or in the opposite direction (negative), is recorded in Relationship Type. For a positive relationship type, an increase (decrease) of the cause leads to an increase (decrease) of the effect; for a negative relationship type, an increase (decrease) of the cause leads to a decrease (increase) of the effect. A causal map ID (cmID) was assigned after all of the 37 generalized cause-and-effect relationships were established. To complete the illustration, a summary and tabulation of the generalized cause-and-effect relationships used to systematically construct the causal map is presented in Fig. 1d, with the contribution of SegmentID 56 highlighted in yellow.

Modifications to the Kim and Andersen protocol [14] for constructing a causal map were motivated by the need to be able to efficiently compare different topics and data segments. Instead of attempting to capture all of the attendant details, data segments were aggregated to highlight the major constructs and the potential mechanisms that connect these constructs. Both the task of manually extracting and organizing the text and the task of generalizing the selected text were time intensive. Yet it was only through multiple and iterative readings of the text that the analyses were effectively framed. This was especially important in generating the causal map, since justifying the underlying logic of relationships required considerable abstraction.

**Fig. 2:**
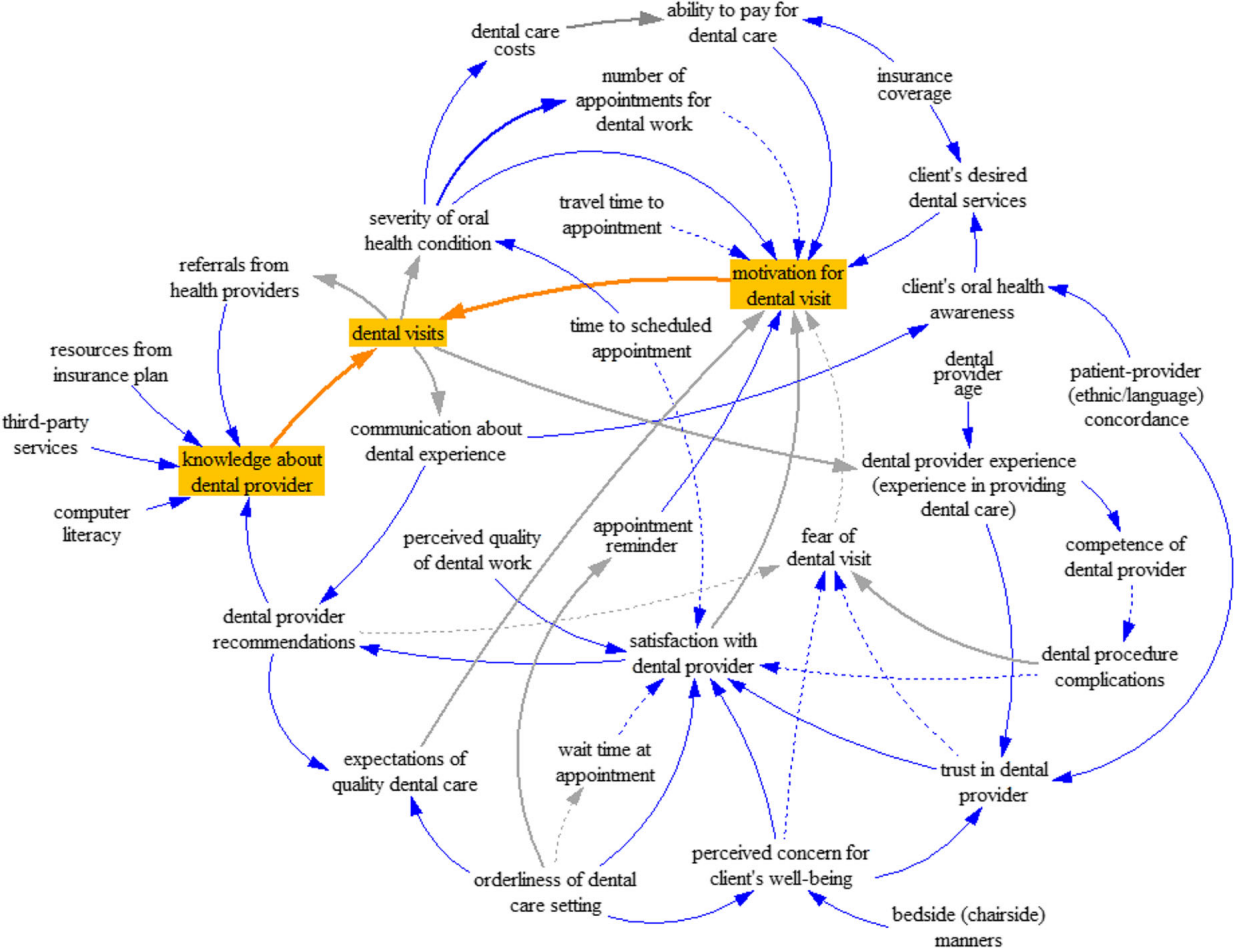
Causal map derived from focus group data. A composite causal map of decisions on where to go for oral health care based on information extracted from focus groups with African American, Dominican, and Puerto Rican older adults. The solid arrows indicate a positive effect (same direction), whereas the dashed arrows indicate a negative effect (opposite direction)

Finally, a composite causal map representing the experience of the patient was systematically constructed to explicate the generalized relationships involved in the decision-making process of dental provider choice (Fig. 2).

These considerations correspond to the access dimensions of acceptability, accommodation, and affordability, i.e., social accessibility to oral health care. Two separate but related issues are manifest: the first involves **finding** dental providers, knowledge about dental providers, and the places where dental providers practice; and the second involves **going** to and returning to dental providers, more specifically, the circumstances at dental practices that motivate dental visits.

The focal variable of the causal map is **dental visits** (highlighted in orange), an indicator of utilization of or realized access to the oral health care system. The two issues of **finding** and **going** to dentists are represented, respectively, as **knowledge about dental provider** and **motivation for dental visit** (also highlighted in orange in the causal map).

The first column in Table 3 lists the 19 variables in the causal map that are included in any of 12 feedback loops involved in finding or going to a dental provider.

**Table 3:**
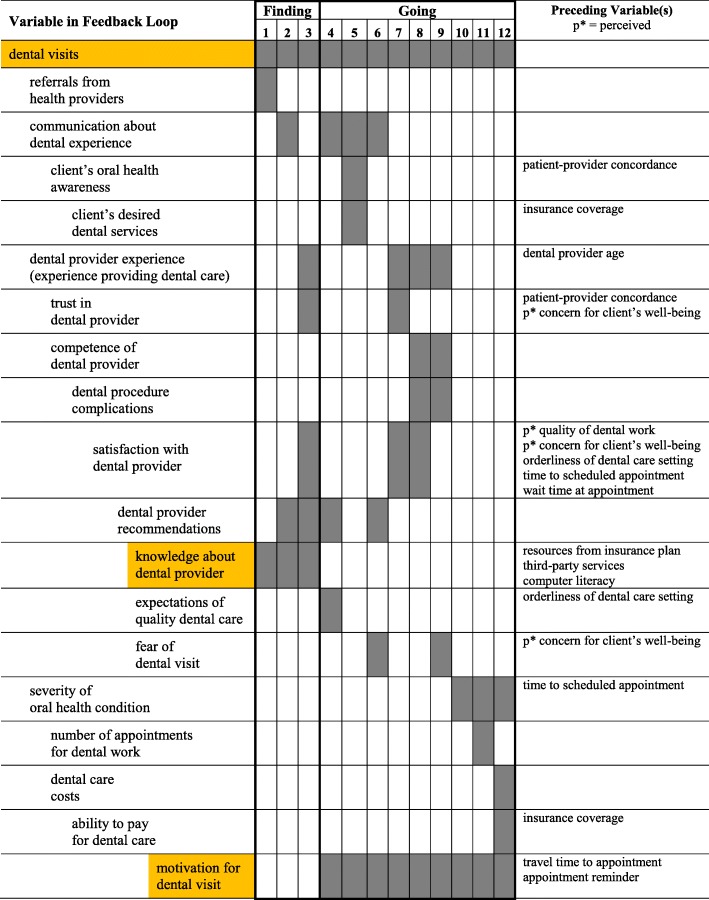
Feedback loops of decisions on where to go for oral health care

All 12 feedback loops include the focal variable **dental visits**. Three feedback loops are associated with **finding** dentists – **knowledge about dental provider** – and 9 feedback loops are associated with **going** to dentists – **motivation for dental visit**. Variables that are not included in any feedback loops are listed in the last column of Table 2 as preceding variable(s), i.e., a cause of the associated variable in the feedback loop.

Below illustrative data segments are presented and discussed, including those related to the example in Fig. 1 (SegmentID 56). Participant responses for how they found dental providers included: resources from insurance plans; phone calls to third-party services; Internet searches; referrals from health care providers; and recommendations from relatives and friends. For example, an African American man explained how he was referred by a dental provider to another dental provider for specialized care: *“So, finally you got a lot of dentists who are really at another level. I’ve been to dentist who have been like, “Listen, I can’t do this, but your union is going to pay.” Mr. [name] is a good dentist for, what do you call it? The root canal!” (focus group 17: African American men with a dental visit in the past year)* Family members and friends were considered to be both important sources of recommendations for **finding** dental providers and influential in **going** to dental visits.

The role of referrals and recommendations in finding dentists are captured in feedback loops 1, 2, and 3; the influence of communication about dental experiences are captured in feedback loops 4, 5, and 6. These feedback loops indicate the potential of using social networks to deliver knowledge and change attitudes and practices towards oral health care. For example, an African American man shared the following remarks: *“She’ll [wife] find a dentist. [pause]. She’ll notice where I’m taking all the pill because it don’t matter to me. I just went to bed. I won’t notice [pause] things like that. But she will. She’s good that way.” (focus group 17: African American men with a dental visit in the past year)* Older adults who lack the support of family and friends may neglect their health and encounter difficulties in obtaining resources to address their health needs, such as information about oral health care options in their neighborhoods and transportation assistance.

According to participants, information and opinions about dental providers and care settings often circulate through word of mouth among community members that may affect their reputations. The following quote from a Dominican man emphasizes the impact of communication in decision-making for selecting dentists:

*“There is something important that I want to explain here, the vast majority of us Hispanics look for our doctors through references, this is very important. In other words if a doctor is good, the neighbor will say…look, so and so is a wonderful dentist. Then we start looking for references. And it’s through the references that we start communicating with one another and we even make an appointment to go see that doctor. Then, if the doctor is bad and did not complete the work well, for this and that reason. It’s always like that, the neighbor will communicate with the second person and that spreads the word. In other words, a job well done will be well received by the community, but people will also know about bad work.” (focus group 15: Dominican men with a dental visit in the past year, translated into English from Spanish)*

Several participants were frightened about going to the dentist either because of fear and the associated pain of dental procedures or the fear of contracting diseases at the dental office. Recommendations from trusted family members and friends may minimize such fears because dental providers and oral health care settings have already been vetted (feedback loop 6). A Dominican woman confidently stated: *“I say, ‘You are afraid? But let me tell you, I have a dentist you will love. Here is the phone number. Here is the phone number. Go to him and you will remember me.’” (focus group 14: Dominican women without a dental visit in the past year, translated into English from Spanish).*

## Discussion

In systems science, both the problems and their solutions are understood as being generated from within the system. Social disparities in oral health result from factors at multiple scales [11]. With regular dental hygiene and professional care, adverse oral health outcomes such as tooth loss may often be prevented. Oral health behaviors and practices are transferred through social relationships throughout the life course, which may either increase or decrease social disparities in oral health. For example, children often adopt the behaviors and practices of their parents. Social relationships among older adults provide mechanisms for the exchange of resources, such as information that is critical in decision-making.

While the life course approach is useful in understanding oral health inequities, there are empirical challenges in testing the proposed theoretical models [36, 37]. Problems with using retrospective studies include the inability to control for exposure times, selection bias due to loss to follow-up, and underrepresentation of racial/ethnic minorities [38]. Agent-based modeling has been proposed as a way forward since it facilitates representation of multiple scales and heterogeneity [39, 40]. Moreover, agent-based models are theory-based and data-driven, and allow for the testing of different plausible mechanisms [41].

A computer model may be used to communicate and learn about the impact of life events and social relationships on oral health. The proposed model design contributes to an existing portfolio of models that have been informed by quantitative and spatial data, as well as the experiences and expertise of research team members [33, 42–46]. Here particular insights from older adults’ experiences that are reflected in focus groups transcripts are included, along with supplementary knowledge about the older adult population in Manhattan and the Bronx. The model is designed to simulate: (1) the life course, i.e., life stages and life events; (2) oral health status, oral health care seeking orientation, and oral health care use; and (3) multiple social relationships as dynamic social networks based on person agent attributes and geographic proximity.

Below are key insights gleaned from the focus groups that are considered important in the model design. First, the inability to pay for oral health care is a significant barrier to accessing services. Second, family members and friends are both important sources of recommendations for finding dental providers and influential in motivating dental visits. Third, participants believe there is a lack of information about oral health in the community and they would like more information about oral health and health care.

The design includes 2 active agent classes: person (older adult, dental provider) and place. Place is further specified into home, work, third place, e.g., senior center, public library, religious institution, and dental clinic [46]. Characteristics of all place agents will include a unique identification number and an indication of status (either open or closed). An additional characteristic of dental clinics is the types of insurance accepted. Further, the identification numbers of older adults who visited each dental clinic and the dates of these visits as well as reminder messages to older adults regarding scheduled appointments will need to be tracked. An additional characteristic of third places is whether health outreach events are held at each location, and if so, the identification number of participants at outreach events, and the participants who needed a referral to a dental clinic affiliated with the outreach events.

According to the focus group transcript analysis, availability (the supply of dental providers) and accessibility (the means of traveling to oral health care) were not perceived to be significant barriers to oral health care. Rather, the inability to pay for oral health care, poor relationships with dental providers, and lack of information on oral health and health care were the major challenges. Therefore, the model design includes 2 types of interventions: (1) social and behavioral interventions; and (2) policy interventions. The first set of modeled interventions would involve community-based outreach education and delivery at places in the neighborhood that provide information, and use different social relationships to direct information and influence changes in oral health care-seeking orientation and oral health care status. The second set of proposed interventions would involve health insurance coverage, specifically, the impact of expanding and ensuring dental insurance coverage throughout the life span, and reducing restrictions to preventive oral health care.

## Conclusions

The findings of this study support the multi-dimensional and multi-level perspective of access to oral health care and affirm a theorized discrepancy in fit between available providers and patients. The presence of resources does not directly translate into use of services by racial/ethnic minority older adults. Despite the relatively high volume of dental providers and the range of transportation options, focus group participants did not believe that their oral health needs were being adequately addressed, whether or not they had recently visited a dentist.

The systematic approach to explicating cause and effect relationships from focus group transcripts introduced here may prove transferable to other research contexts. The product of this approach, a causal map, provides a visual representation of major factors and relationships involved in the decision-making process.

From both epistemological and ontological standpoints, however, system dynamics involves more than the mechanics of creating a causal map. Rather, there is a philosophical understanding that in order to solve large, complex problems, it is important and effective to consider the needs of others [47]. The ability to incorporate qualitative data into a causal map allows direct inclusion of the views of underrepresented populations into the hypothesized cause and effect mechanisms explicated.

The lack of information about oral health may be compromising the use and quality of oral health care among racial/ethnic minority older adults. This finding is consistent with key informant views that senior center attendees did not regard oral health concerns with the same degree of immediacy as high blood pressure (indicative of hypertension) or high blood sugar (indicative of diabetes) [48]. Well-informed community members may fill critical roles in oral health promotion, as they are viewed as highly credible sources of information and recommendations to dental providers. Disseminating up-to-date information at frequented sites to older adults and the community at large about the importance of oral health, proper dental hygiene practices, and local oral health care options remain public health priorities.

## Additional file

Additional file 1: Focused Group Interview Topic Guide. A topic guide consisting of open-ended questions that was developed by the research team and used by the focus group moderators to facilitate the sharing of personal experiences among participants regarding reasons why people may or may not visit a dentist. (PDF 57 kb)

## Abbreviations

cmCause: Causal map cause
cmEffect: Causal map effect
cmID: Causal map identification number
dsCause: Data segment cause
dsEffect: Data segment effect
SegmentID: Data segment identification number
