# Supplementary material for: Using focus groups to design systems science models that promote oral health equity
Source: BMC Oral Health. 2018 Jun 4;18:99. doi: 10.1186/s12903-018-0560-0 (PMC5987593; doi:10.1186/s12903-018-0560-0)
Supplement: Supplementary file 1 — Focused Group Interview Topic Guide. A topic guide consisting of open-ended questions that was developed by the research team and used by the focus group moderators to facilitate the sharing of personal experiences among participants regarding reasons why people may or may not visit a dentist. (PDF 57 kb) [file 12903_2018_560_MOESM1_ESM.pdf]

Integrating Social and Systems Science Approaches to Promote Oral Health Equity

Focused Group Interview Topic Guide

First, I would like to thank everyone for meeting with us today. My name is ____, and this is _____ (Name of RA), and we are with [Columbia University / NYU]. At today's meeting, we would like to discuss as a group reasons why some people may or may not go see a dentist. We are interested in hearing not only your own opinions and experiences, but also about the reasons why other seniors in your community may or may not go see a dentist as well.

1. Senior Centers
    a. Why do people go to senior centers?
    b. What kinds of services or programs do senior centers provide for people?
    c. What kinds of health-related services or programs do senior centers provide?

2. Oral Health
    a. What do you think of when we say "oral health"? (Probe for what seniors more generally think)
    b. What do you think seniors need to do to have a healthy mouth?
    c. How important is oral healthcare is for [men/women] of your age? Why or why not?

3. Going to the Dentist
    a. How often do you and other seniors in your community go to the dentist?
    b. How often should people your age go? Why?
    c. What things lead seniors to go to the dentist?
    d. What things lead seniors to <u>not</u> go to the dentist?
    e. What things lead some people to go more than others?

4. Recent Dental Symptoms
    a. What are the most common types of oral health problems people your age experience?
    b. What can people do to prevent problems? (Probe: self care, nutrition, dentist visits, etc)
    c. Do you think there is anything a dentist can do to help prevent oral health problems?
    d. Do you think there is anything a dentist can do to cure oral health problems?

5. Perceptions of Dentists
    a. What do seniors in your community think about dentists? Why?
    b. What are the characteristics of a good dentist? A not good dentist?

6. Dental Fears
    a. Do you think some people your age are afraid of going to the dentist?
    b. What kinds of things about a dental visit are people scared of?
    c. Do you think that these fears keep people from going to the dentist when they have an oral health problem?
    d. Do you think that these fears keep people from going to the dentist for a regular cleaning or exam?

Interpersonal Factors:

7. Family
    a. Do people your age talk with their families about going to the dentist? Probe: Children? Spouses?
    b. Is there anything families do that discourages or prevents seniors from going to the dentist?
    c. Is there anything that families do to encourage or help seniors go to the dentist? (Probe: Financial, Transport)

8. Peers
    a. Do people your age talk with their friends about going to the dentist?
    b. Is there anything friends do that discourages or prevents seniors from going to the dentist?
    c. Is there anything that friends do to encourage or help seniors go to the dentist?

9. Other Neighborhood Institutions
    a. Do people your age talk to people in your church about going to the dentist?
    b. Is there anything that people in your church do or could do to encourage or help seniors go to a dentist?
    c. Do social service organizations every talk to people your age about going to the dentist?
    d. Is there anything that social service organizations do or could do to encourage or help seniors go to the dentist? (Probe: Information, Referrals, Transport, Financial Assistance)

Community-Level Factors:

Next I would like to talk about the neighborhoods in which you live and how that might influence whether or not seniors visit the dentist.

10. Availability of Dentists
    a. Are there any dentists or dental clinics in your neighborhood? (Probe: Signs? Ads?)
        i. Do you think people in your neighborhood go to this dentist or dental clinic? Why or why not?
    b. Has there ever been a dentist or dental clinic in your neighborhood? If not, Why not? What happened to it?
    c. How do people in your neighborhood find a dentist?
    d. What is important in deciding where to go?
    e. Is there anything about your neighborhood which makes it <u>difficult</u> for seniors to go to the dentist?
        i. Probe: Anything about buildings? Sidewalks? Neighborhood safety?
    f. Anything about your neighborhood that makes it easier for seniors to go to the dentist?

11. Language
    a. Do you think people your age who only or predominantly speak Spanish have difficulty finding information about dental care?
    b. Do you think seniors who only or predominantly speak Spanish have difficulty finding a dentist who also speaks Spanish?
    c. Do you think the lack of Spanish language information or Spanish speaking dentist might prevent or discourage some seniors from going to the dentist?
    d. Do you think that documentation status might prevent or discourage some seniors from going to the dentist?

12. Lack of Affordable Dental Care
    a. Do people your age see dental care as something they can afford? Why or why not?
    b. Can seniors find a dentist that is affordable?
    c. Do seniors have insurance to help them pay for a dentist?
    d. Do seniors know that Medicaid and Medicare can help pay for a dentist?
    e. Can seniors find a dentist that accepts insurance?
    f. Do seniors know of others (family, friends) who could help pay for dental care if they asked them?
    g. Do seniors know of any resources or services that could help you pay for dental care?
    h. Does a lack of affordable dental care cause seniors to not go the dentist?

13. Transportation

a. Do seniors have a way of getting to a dentist's office?
b. Is lack of transportation a reason for not going to the dentist? How important of a reason is this?
c. Do seniors have family, friends, or community organizations that could help them get to the dentist if they asked?

14. Eldersmile
    a. Many senior centers in {Neighborhood} have a program called Eldersmile in which dentists come to the senior center. Do the Eldersmile dentists ever visit your senior center?
    b. Do you all know what the Eldersmile dentists do?
    c. What have people told you about an Eldersmile exam?
    d. Would seniors be willing to have an Eldersmile dentist examined them? If not, Why not?
    e. Of those who have been examined, why do you think they decided to go for the Eldersmile exam?
    f. Of those who have not been examined, why do you think they have not gone for the Eldersmile exam?

15. Hypertension and Diabetes
    a. In addition to dental care, seniors also often need to regularly monitor their blood sugar and their blood pressure. How important do you think it is for seniors to get your blood sugar and blood pressure checked regularly?
    b. Where do seniors go to get their blood sugar or their blood pressure checked?
    c. Would seniors be willing to have their blood sugar or their blood pressure checked when they visited dentist's office?
    d. What would seniors like you like or not like about having their blood sugar or blood pressure checked as part of their dental visit?
    e. If the dentist were to provide seniors with a blood sugar and blood pressure check as part of your dental visit, do you think this would this make people more likely or less likely to go to the dentist? Why or why not?

Wrapping Up:

16. Okay, we have discussed a lot of different things that lead seniors to not visit the dentist. Thinking of all of the things you have heard and discussed here today, what do you think is the most important reason for why people like you do not go to the dentist?

17. One of the goals of our meeting today is to help seniors go to the dentist regularly. If you were in charge of designing a program for seniors, with all the money and resources you wanted, what would it offer?
    a. Where would it be located?
